# Perspectives of Individuals With Long COVID on Virtual Physical Rehabilitation: A Qualitative Study

**DOI:** 10.1016/j.arrct.2025.100526

**Published:** 2025-09-18

**Authors:** Kriti Agarwal, Catherine M. Tansey, Amanda K. Rizk, Marla K. Beauchamp, Bryan A. Ross, Jean Bourbeau, Maria Sedeno, Laura Barreto, Rebecca Zucco, Emily Crowley, Tania Janaudis-Ferreira

**Affiliations:** a School of Physical and Occupational Therapy, McGill University, Montreal, Québec, Canada.; b Respiratory Epidemiology and Clinical Research Unit, Centre for Outcomes Research and Evaluation, Research Institute of McGill University Health Centre, Montreal, Québec, Canada.; c School of Rehabilitation Science, McMaster University, Hamilton, Ontario, Canada.; d Willkin Health, Montreal, Québec, Canada.

**Keywords:** Exercise, Long COVID, Physical rehabilitation, Qualitative, Rehabilitation, Virtual Rehabilitation

## Abstract

**Objective:**

Coronavirus disease 2019 is an infectious disease caused by the severe acute respiratory syndrome coronavirus 2, and long COVID is a chronic condition characterized by symptoms persisting for atleast 3 months after infection. To explore the perspectives of individuals with long COVID after an 8-week virtual physical rehabilitation program.

**Design:**

Qualitative descriptive study.

**Setting:**

Clinics and research cohorts.

**Participants:**

Adults (n=132) with confirmed or probable COVID-19 infection and persistent symptoms, including reduced mobility, muscle weakness, dyspnea, and/or fatigue, were recruited in a randomized controlled trial. Thirteen intervention group participants who completed the rehabilitation program were included in this qualitative study.

**Interventions:**

The intervention group (n=65) received 8 weeks of tailored, symptom-titrated exercises, weekly educational sessions, and usual care, whereas the control group (n=67) received only usual care.

**Main Outcome Measures:**

Semistructured videoconference interviews were conducted and analyzed using deductive thematic analysis.

**Results:**

Participants’ age (mean ± SD) was 48.3±15.6 years, 6 had been hospitalized during their COVID-19 infection, and the duration of long COVID (mean ± SD) was 18.8±7.2 months. Four themes were identified: (1) Motivation and confidence: most participants expressed confidence in joining the program, motivated by health goals, scientific contribution, and reassurance from professional support. (2) Program features: the program was praised for its well-organized format, ideal duration, convenient scheduling, supportive kinesiologists, and individualized exercise plans. (3) Health effects: while most reported physical and emotional improvements (eg, increased energy, mobility, and confidence), some noted challenges upon returning to work. (4) Post-program suggestions: participants intended to continue exercising but faced barriers such as fatigue and a lack of motivation, highlighting the need for continued support and resources to maintain progress.

**Conclusions:**

This study highlights the positive effects and relevant challenges associated with completing an 8-week personalized, symptom-titrated virtual physical rehabilitation program for individuals with long COVID, emphasizing the need for tailored support and ongoing resources to facilitate sustained recovery.

Coronavirus disease 2019 (COVID-19) is an infectious disease caused by the severe acute respiratory syndrome coronavirus 2 (SARS-CoV-2). Long COVID is a chronic condition resulting from SARS-CoV-2 infection, characterized by symptoms that persist for at least 3 months and present in a continuous, relapsing-remitting, or progressive pattern, often affecting multiple organ systems.^1^ The condition is characterized by more than 200 reported symptoms, with fatigue, cognitive dysfunction, and shortness of breath among the most prevalent.^2^, ^3^, ^4^ These symptoms can be triggered or worsened by minimal physical or cognitive exertion,^4^^,^^5^ leading to substantial impairment of functional capacity and quality of life.^2^^,^^6^

Because of the limited number of randomized controlled trials (RCTs) on the physical rehabilitation of long COVID, health care professionals lack evidence-based guidelines for the management of long COVID.^7^ Our team conducted an RCT,^8^ which offered an 8-week virtual physical rehabilitation to individuals with long COVID who experienced fatigue, dyspnea, muscle weakness, and/or reduced mobility. This was the first trial to test a fully virtual, individualized, symptom-titrated exercise intervention with careful monitoring of adverse events, including those related to post-exertional malaise (PEM),^4^^,^^5^ a common and debilitating condition associated with long COVID. The intervention group showed higher adherence to supervised exercise sessions (96%) compared with independent sessions (74%), suggesting that supervision may have supported engagement in exercise interventions. These participants also showed greater improvements in health status and symptoms compared with the control group.^8^

Qualitative interviews in clinical trials^9^ provide valuable insights into patient perspectives, treatment experiences, and perceived benefits. Considering the wide variation in the clinical manifestations of long COVID, the limited understanding of this relatively new condition,^10^ the scarcity of rehabilitation programs,^10^^,^^11^ and the ongoing debate regarding optimal rehabilitation approaches,^10^^,^^12^ qualitative interviews hold particular value in this clinical context.

This study built on the quantitative findings from the parental RCT and aimed to explore the perspectives of individuals with long COVID on their experience with the virtual physical rehabilitation program. We sought to address the following research questions: (1) how do participants perceive the 8-week virtual physical rehabilitation program, particularly in terms of motivation for participation, the program’s structure, and any encountered barriers? and (2) what are their recommendations for improving the program?

## Methods

### Research team and reflexivity

The interviews were conducted by an experienced, bilingual woman research associate (C.M.T.), a PhD holder with expertise in qualitative methods, who was independent of the intervention team. There was no prior relationship between C.M.T. and the participants.

### Study design

This qualitative descriptive^13^, ^14^, ^15^ study considered a subgroup of individuals with long COVID who completed a virtual physical rehabilitation intervention of an RCT.^8^

The study followed the Consolidated Criteria for Reporting Qualitative Research checklist (supplemental appendix S1, available online only at http://www.archives-pmr.org/).^16^ Ethics approval was obtained from the Research Institute of McGill University Health Centre and McMaster University, Canada.

#### Description of the intervention

The RCT^8^ included 132 individuals with long COVID, who were randomized to either the intervention or control group. The intervention group (n=65) received an 8-week virtual physical rehabilitation program consisting of one-on-one, personalized, symptom-titrated functional aerobic and strengthening exercises, weekly educational sessions, and usual care. Exercise sessions were delivered 3 times per week, and individuals followed a progressive phase-out design, beginning with supervised Zoom sessions and gradually transitioning to independent sessions, for a total of 14 supervised and 10 independent sessions, each lasting 45-50 minutes. The control group (n=67) received only usual care. Details about the rehabilitation program have been previously reported.^8^

#### Participant selection

The RCT included adults with a history of COVID-19 infection who lived with self-reported persisting symptoms of fatigue, dyspnea, reduced mobility, and/or muscle weakness in the provinces of Quebec or Ontario, and who did not suffer from any uncontrolled or severe illness that would preclude exercise.^8^

A research associate emailed participants who had initially consented to interviews during the RCT consent period. The target sample size was 12-15 participants or until data saturation was achieved.^17^^,^^18^ Purposive sampling was employed to ensure variation in age, sex, language preference, and date of study (RCT) entry. Fourteen individuals were invited; 1 was later excluded because of an availability issue, resulting in the enrollment of 13 participants. No incentives were offered. Verbal consent was obtained and recorded at the start of each interview.

#### Setting and data collection

Participants were interviewed via a videoconferencing platform in either English or French. One-on-one interviews were conducted between the participant and C.M.T. following an open-ended format guided by the Theoretical Domains Framework (TDF)^19^^,^^20^ (supplemental appendix S2, available online only at http://www.archives-pmr.org/). Interviews lasted an average of 27.2 minutes and were digitally audio- and video-recorded. No field notes were taken, and no repeat interviews were conducted. Interview recordings were transcribed verbatim, with all transcripts deidentified. French interviews were simultaneously translated and transcribed into English by a professional transcriptionist. All transcripts were reviewed for accuracy by C.M.T. to enhance descriptive validity.^21^ Data saturation was assessed through iterative transcript review, with no new themes emerging after the 12th interview.^22^ Transcripts were not returned to participants for review or corrections.

### Data analysis

A hybrid thematic analysis was conducted, combining both deductive and inductive approaches. Interview questions were informed by the TDF, providing a deductive structure. Data analysis followed Braun and Clarke’s^23^ 6-phase process, allowing for inductive theme development. Two researchers (K.A. and C.M.T.) familiarized themselves with the data, independently generated initial codes, and collaboratively identified potential themes in consultation with the principal investigator (T.J.-F.). Themes were then reviewed for coherence, consistency, and distinctiveness before being clearly defined and named. Thematic mapping was used to further refine and validate themes across the dataset. To facilitate the analytical process, Dedoose software (version 9.2.12 for Mac) was used.^24^^,a^ Participant feedback on the findings was not obtained.

## Results

### Patient characteristics

Participants age (mean ± SD) was 48.3±15.6 years. Six out of 13 participants were hospitalized during their COVID-19 infection. At the time of recruitment, the duration of long COVID (mean ± SD) was 18.8±7.2 months, and most (n=12) participants reported experiencing PEM. Interviews were conducted at (mean ± SD) 32.3±20.6 days after the intervention. Detailed participant characteristics are provided in table 1.

**Table 1:** Characteristics and interview details of the study participants.

Characteristics	Participants, n (%)
**Age (years), median (IQR)**	54 (31, 59)
**Sex**	
Men	6 (46)
Women	7 (54)
**Level of education**	
High school	3 (23)
College	3 (23)
Undergraduate	5 (38)
Graduate	2 (15)
**Participants hospitalized during their COVID-19 infection**	6 (46)
**Presence of PEM at study entry (DePaul PEM questionnaire)**	
Yes	12 (92)
No	1 (8)
**Duration (months) of long COVID to randomization into the intervention, median (IQR)**	20 (15, 21)
**Exercise sessions attended with kinesiologists**	14 (100)
**No. of adverse events reported during the program**	
None	3 (23)
1	7 (54)
2	2 (15)
3	1 (8)
**Language preference for the interview**	
French	7 (54)
English	6 (46)
**Length (minutes) of interview, median (IQR)**	28 (25, 32)

### Qualitative interviews

Four overarching themes were generated from the dataset: motivation and confidence, program features, health effects, and post-program suggestions. These themes were both predefined, drawing on existing theories, and emergent, identified through an inductive analytical approach.

#### Motivation and confidence

Participants’ reasons for joining the trial were multifaceted. For some, the main motivation was to deepen their understanding of their newly diagnosed health condition; others needed to share their emotions amid limited long COVID services. A few hoped to improve their symptoms, enhance their physical health, access care through the trial, and contribute to research that supports others facing similar challenges.

*“I wanted to be among the first to recover from long COVID, but I also wanted to contribute to science, be part of the group that could move things forward to help others.”* (Participant 13)

As the same (participant 13) stated, *“I’m the lucky one,”* a sense of gratitude was reflected. Similar feelings of happiness, enthusiasm, and optimism about being assigned to the intervention group were expressed by all participants. Many also shared that family and friends were supportive and hopeful about their participation.

Many participants expressed confidence in exercising at home and readiness to join the rehabilitation program. Although most did not fear home-based exercise, a few emphasized that they needed professional guidance and encouragement to exercise safely and effectively, highlighting the value of structured supervision. One (participant 11) expressed reassurance that the program avoided medications or potentially harmful interventions, explaining, *“…what encouraged me was knowing that the people leading the program were kinesiologists, or movement scientists so I wasn’t going to be launched into something that would be dangerous for me.”*

While participants were generally enthusiastic, several expressed initial concerns. These included doubts about their ability to perform exercises, fear of symptom exacerbation, the time commitment required, and general nervousness about starting the program. In a closely related sentiment, one (participant 9) mentioned, *“I felt nervous because I had no idea how easy or how tough it is to participate.”*

#### Program features: program format

Participants widely appreciated the program’s structure, highlighting the frequency (3 times/week) and duration (40-45 minutes) of the exercise sessions as manageable and considerate of other commitments. The convenience of the virtual format and minimal equipment (eg, chairs, existing weights, household items like cans) was also valued, with no concerns reported regarding space, safety, or technology use.

*“…(exercise session) was 40 minutes; I feel that was a pretty good amount of time… the three times a week was probably my favorite point, because I had something to do.”* (Participant 10)

Despite overall satisfaction, 3 participants suggested reducing the session frequency to twice weekly to better accommodate their return to work or effectively monitor PEM. Two participants reported cognitive fatigue from virtual sessions and preferred in-person options for more direct supervision. Some described challenges in integrating sessions into their schedules because of limited space.

*“To be honest, more than two (sessions/week) is difficult, because I don’t have time to assess my PEM, usually occurs 48-72 hours after my activity. So, with three, well it was hard to see if I had PEM or not because I was always on to the next.”* (Participant 8)

Although the 8-week program was well-received, most participants recommended extending it by 4 weeks to support continued progress and routine building.

#### Program features: kinesiologist supervision and independent sessions

Strong appreciation was expressed for the kinesiologists’ supervision, practical guidance, and encouragement to exercise within limits, with their assistive, cheerful, and adaptable approach, as well as their availability outside work hours, which enhanced participants’ confidence and performance. Most participants found the nature, type (aerobic, stretching, or strengthening), and difficulty level of the exercises to be *“perfect,”* highlighting progression and variation as key motivators for sustained engagement.

*“…(the kinesiologist) always kept the exercise just challenging enough to give me the impression of progressing, but not too challenging that I could not complete them or felt overtired.”* (Participant 5)

Several participants described feeling *“energized”* immediately after sessions, with some noticing an improvement in their energy over the course of the program.

*“At the beginning of the program, it felt like my PEM was activated. But as time went on, I felt like I was sleeping better, so I had more energy, and at the end, I had less PEM because of the progression throughout.”* (Participant 7)

Others reported experiencing fatigue immediately after exercise, typically lasting <3 hours. To manage this, a few participants structured their schedules to avoid overexertion, while others were able to resume their daily activities after taking a short rest. For several participants, these strategies evolved over the course of the program (table 2). One (participant 12) reported experiencing PEM if engaging in activities after the session. Similarly, another (participant 6) stated, *“They were fatiguing but I felt good doing them, because of that, it was fine. If I did other things afterwards, that’s where I really started to feel the fatigue.”*

**Table 2:** Additional participant quotations categorized by themes.

Themes	Participant’s Quotes
**Motivation and confidence**	
Concerns before joining	“I had some concerns, yes because I frequently have PEM, so I was wondering if it would be set off and worried about what I’d do if it did happen, but my questions were answered.” (Participant 8)
	“At the beginning, I don’t know to be honest. But I feel like any information, or anything may help at that point. I was a little bit skeptical to begin with.” (Participant 9)
	“I did have a lot of knowledge about long COVID, but I was on my own, so I wasn’t too sure how to set myself up.” (Participant 12)
Reasons for joining	“I accepted because I found the approach was interesting. In my case, my main symptom was fatigue and muscle weakness, so I thought it was a good opportunity to at the very least improve my condition.” (Participant 7)
	“Because long COVID hasn’t been around for very long, and I think we need help. There are so many people who have this problem and there’s not much currently known about it. So, if nobody participates in this type of project, we’ll never see any improvement.” (Participant 8)
	“I think I suffer from the long COVID symptoms and of course from the media I know that I am not alone. It’s a lot of people… I feel like if anything can help with anyone else similar to my situation, I would be happy to do it.” (Participant 9)
	“I accepted with the hopes of helping move the research forward and better the understanding and find ways to solve the problem.” (Participant 12)
Motivations	“My wife, she was just as anxious as I was to try to do exercises, even if the study was going, to give me hints to be able to help me” (Participant 6)
	“I was happy because, in my situation, I didn’t really know where, or whom to turn to for help with regards to how to get better. So, when I found out that I could be part of the study, I was happy. I felt like I was being looked after.” (Participant 7)
	“I was excited. I was hoping that it could potentially help with some of my symptoms I was experiencing. Yeah, overall, just excited to be a part of it.” (Participant 10)
	“I was really hoping to be in the exercise program group because I suffered so much from long COVID symptoms, I had been trying to improve my condition for three years, so I really hoped to be selected for it.” (Participant 11)
**Program features**	
Program format	“It was a nice length, yeah. It wasn't too long that I was getting bored of it or anything, but it was still long enough that I exercised enough that I would need a shower after.” (Participant 4)
	“I found that is very helpful when you have one-to-one instead of a big group… In big group, I don’t have the courage to ask questions, right? But one-to-one, I have the freedom to say, I don’t understand what it means. So, she would demo, and that is very, very helpful to us.” (Participant 9)
	“*[the virtual sessions were not challenging]* because it meant I didn’t have to travel, which would have been a challenge for me.” (Participant 12)
	“I think it was well done, except maybe at the end when we wound up with one session per week, I may have preferred having a slightly longer session so she could explain things more, support me a bit more for the independent sessions.” (Participant 13)
Kinesiologist supervision	“I learned a few new exercises. I felt good after each session because it kind of stimulates my body, makes me feel good about myself.” (Participant 4)
	“I was fortunate to be assigned and extremely helpful, dynamic, versatile, and cheerful kinesiologist. She was quick to adapt to my evolving needs, and always found exercises that challenged me without pushing me beyond my limits. My sessions with her became the highlight of the week throughout the duration of the program.” (Participant 5)
	“I had to pace myself because I tend to be too enthusiastic for my capacity, but I liked that we started so gradually. We started with seated exercises.” (Participant 8)
	“Have been bit worn out for half an hour, 45 minutes, that required a bit of recuperation but… I could continue on with my other daily things.” (Participant 8)
	“I experienced it during our first or second meeting, the exercises threw everything off. We had to modify the program because it was too much for me… She adapted very well to my condition and how I reacted to the exercises.” (Participant 8)
	“To start with, every time after class I have to designate myself, like, two three hours just do nothing to recover. But toward the end of the program, I think about week five on, I already started to feel ah ha, I finished the session, half an hour relaxes, and I can get back to my kind of life.” (Participant 9)
	“Generally, I was a bit fatigued from it, but I was still able to get up and do stuff around the house. But generally, yeah, my energy levels were a bit lower, sometimes I’d have a bit of muscle pain, stuff like that.” (Participant 10)
	“If I hadn’t had that frequency of supervised sessions with *[the kinesiologists]* at the beginning that I would have been very discouraged. I wouldn’t have been motivated to train, but discouragement also about the level of difficulty I was experiencing regarding understanding the exercises, so I would have been scared of sustaining an injury.” (Participant 11)
	“What I also liked was that when the kinesiologist saw that I was starting to struggle, malaise was beginning and I struggled to concentrate, she would interrupt the session and say, OK we’re going to stop there so you don’t wind up feeling worse.” (Participant 13)
Independent session	“*[The kinesiologist]* and I would be reviewing the independent session together, there was a motivation there to do the exercises, and do them well, so I didn’t miss one.” (Participant 1)
	“I think I pushed myself a little bit more. *[The kinesiologist]* was able to pace me, when we were doing exercises. So, when I was alone… I pushed myself to finish it.” (Participant 6)
	“I found that the transition to independent session was very quick *(chuckling)*. It’s less motivating to do the exercises independently, to make it part of our day. When I was already tired and I knew I had a session with her, it was motivating. But when I was alone… that was harder.” (Participant 8)
	“Another big part of my long COVID is I have anxiety and depression, and that affects my mood obviously, and my motivation, energy levels, all of that.” (Participant 10)
Educational session	“I did pay attention to the material, did the readings. But for me, that wasn’t the major component of the program... if that wasn’t there, I could have done another exercise.” (Participant 1)
	“I think all the aspects of the physical impacts of post-COVID were addressed, as well as the psychological impacts. So no, I think it was very well made.” (Participant 5)
	“I felt like the eight topics were very important. If it was longer, say over 10 weeks, maybe it would be OK to add some in, but I think all the topics covered were important to cover.” (Participant 13)
**Health effects**	
	“And I still feel like … you are progressing, and you are gaining something and that’s extremely encouraging and gives you hope that things will eventually get to a situation that might not be exactly like it was before, but at least livable.” (Participant 5)
	“I was hoping to get to have a better resistance to endurance exercise, but it’s improved somewhat. Not as much as I had hoped to, but I think it’s… from what I understand of the condition now, it’s what to be expected.” (Participant 5)
	“...at the beginning of the program, I did the sessions at the beginning of the day, and I felt out of energy for the rest of the day. But as we moved forwards through the program, I felt that while I had consumed a portion of my energy, I did still have enough left over to continue with my day. So, there was an evolution there as time went on.” (Participant 7)
	“*[Baseline symptoms]* did not improve much, unfortunately. I’m a bit stronger in my arms, maybe, but I’m no better at going up stairs” (Participant 8)
	“Actually every week, the difference from every week is very obvious. Before the program, I could not talk to you like this. I would need to stop to cough. Even when I started the first session, I kept coughing a lot. Now I manage to talk to you without being interrupted with coughing. Because talking is sometimes difficult, but now, I gained back, not only the confidence in myself like the exercise. The program is giving me the physical and emotional kind of like get back to life.” (Participant 9)
	“When I was doing the program, my muscle pain and shortness of breath were improved a decent amount, a noticeable amount I would say… I did have increased anxiety because of the long COVID, that was helped a good amount.” (Participant 10)
	“I never thought I would regain that ability. So, it really was life-changing and what I want to do is continue training like this even if it’s really difficult for me to fit it in my schedule three times a week.” (Participant 11)
**Post-program suggestions**	
	“I would say the program is REALLY good but after this program, if we can have the similar, like long COVID patients to do in a group section, like a virtual group.” (Participant 9)
	“Patients suffering from long COVID often receive general guidance, like be active, do this, do that, but there isn’t necessarily any follow-up or support. And for me, that’s what the program gave me, and that’s why I think I saw changes. So yes, I would strongly recommend it and I’d like doctors to be better equipped and be able to refer patients… I’d like to see it be integrated into clinical practice because I think it works.” (Participant 11)
	“it would be interesting being an individual within a small group that exercises together. Say with people who are at your same level… being able to cheer each other… But you can’t really do it with someone who doesn’t have long COVID because the way it’s perceived is that if you don’t show up, it’s because you’re a bit of a failure. But are you really a failure or are just maxed out and you don’t have any energy because your day was too demanding? And people don’t necessarily understand that.” (Participant 12)

The independent sessions were described as *“flexible”* and *“easy,”* with participants expressing confidence in repeating exercises alone and valuing the option to contact kinesiologists when needed. However, nearly all participants faced challenges in sustaining routines alongside other commitments, managing fatigue upon returning to work, maintaining motivation, and exercising independently, expressing a desire for ongoing support and encouragement from a kinesiologist.

#### Program features: educational sessions

The weekly educational sessions were perceived as important, with the content being interesting and informative. The concise and focused format was also well-received, as one participant expressed, *“It was the perfect format, not too long, to the point and applicable to day-to-day life.”* (Participant 5)

These sessions were highly valued for their practical guidance, especially in managing long COVID symptoms such as PEM, *“Helping us learn about when we’re pushing it, reaching our limit, so we don’t wind up like I did after the first few sessions having PEM.”* (Participant 8)

Some expressed a desire for in-depth information on topics such as anxiety management, reasons behind symptom fluctuation, pacing strategies, and navigating the health care system.

#### Health effects

Participants reported a wide range of improvements attributed to the program. Many described increased energy levels, greater confidence in exercising, reduced stress, and feeling less concerned about their condition. They also reported a deeper understanding of their symptoms, particularly in managing PEM.

Physical and functional improvements were also frequently mentioned, including enhanced endurance and mobility. Participants shared tangible examples such as *“taking longer walks with my dog” and “no longer needing a cane to walk.”*

*“I’m more mobile, in stairs. My endurance for walking is better, so it’s all positive.”* (Participant 1)

The program’s effect extended beyond physical benefits. Several participants reported improvements in cough, fatigue, shortness of breath, muscle pain, anxiety, and depression. Others mentioned relief from chronic issues, such as nerve compression associated with prior COVID-19 hospitalization.

*“This program helped me to get not only physical but emotional, the depression is kind of SO scary to think about… I didn’t want to discuss about COVID at all for a long time, but the program got me to look at it.”* (Participant 9)

Some participants also mentioned the progression of their overall health, *“I start the program at my health stage, it was about 4 out of 10, right now I would have to say about 7 out of 10.”* (Participant 9)

However, a few reported no change in their symptoms. One participant noted a deterioration upon returning to work, *“With the program, my symptoms remained pretty much the same. I didn’t necessarily see any improvement over the summer… since going back to work, my condition has deteriorated.”* (Participant 12)

#### Post-program suggestions

All participants expressed a strong intention to continue exercising after the completion of the program. Some were motivated by the benefits they had gained, while others were driven by a fear of losing their strength and the progress they had made.

*“…because of the program, I will include exercise sessions at home in my routine because I’ve seen the benefits of that.”* (Participant 7)

However, several participants reported difficulties in maintaining their exercise routine, citing issues such as a lack of motivation or discipline, fatigue upon returning to work, fear of symptom relapse, and a lack of supervision or variation in the exercises. One (participant 12) shared, *“Desire is there, but energy is not really there.”*

Another participant expressed, *“I just wish I had more contact with the project to get some more information, moving further into my recovery.”* (Participant 9)

All participants supported the idea of recommending the program to others, highlighting its value in educating and assisting individuals in managing long COVID and its symptoms.

*“This program is really helpful to share… sometimes the physician doesn't know how to work with the symptoms. And you guys helped me…”* (Participant 3)

Some participants provided valuable suggestions for improving the intervention, including creating a small social group for individuals with long COVID to exercise together, ensuring the continuation of the program (*“if there’s ever a phase 2”*), and making it more widely accessible. They also recommended offering additional resources, such as weekly exercise recordings, to help maintain motivation and adherence.

Additional quotes for each theme are presented in table 2.

## Discussion

An overarching message emerging from this qualitative study was that participants regarded the rehabilitation program as both essential and beneficial. They highlighted the importance of structured, closely supervised support tailored to their physical and personal needs, which was deemed crucial for building confidence, exercising safely, and making effective progress. The program was also perceived to alleviate symptoms, reduce stress, and enhance understanding of self-management strategies. A shared interest in program extension or a second phase further underscored the value participants place on continued guidance for sustained improvement. These qualitative insights are consistent with the parental RCT,^8^ which demonstrated significant improvements in dyspnea, fatigue, pain, discomfort, and overall perceived health among the intervention group participants.

Many participants joined the trial seeking structured support, a deeper understanding of their condition, access to informed health care guidance, and a safe space to voice their concerns, reflecting gaps in long COVID clinical management during the trial period.^25^, ^26^, ^27^ These motivations may have been influenced by broader systemic barriers, including limited access to care, poor communication with health care providers, and a lack of provider empathy,^27^, ^28^, ^29^ making recovery an isolating experience.^30^ Although some initially expressed concerns about symptom exacerbation or their ability to exercise, most enjoyed the program and remained hopeful it would provide meaningful support. This was reflected by a low drop-out rate (6%) in the intervention arm of the parental RCT.^8^

A personalized approach and symptom management guidance were key to addressing the evolving needs of the participants. These findings align with prior COVID-19 rehabilitation studies,^30^ highlighting the educational component as a vital space for shared reflection. While adherence in the intervention arm of the RCT^8^ was high for both supervised (96%) and independent (74%) sessions, participants found the independent sessions more challenging because of a lack of motivation and direct supervision, suggesting that structured guidance remains crucial for sustained engagement.

Participants reported multifaceted improvements from the program, including enhanced self-management strategies, such as pacing and recognizing energy limitations, to manage PEM. Many noted gradual improvements in their energy, daily performance, and overall health status throughout the intervention. These experiences are consistent with existing research highlighting the positive effects of physical rehabilitation and structured self-management interventions on mental health and health-related quality of life.^31^, ^32^, ^33^

Despite these improvements, some participants experienced challenges, including persistent symptoms, post-exercise discomfort, and difficulty balancing rehabilitation with personal responsibilities. Similar challenges were reflected in the parental RCT,^8^ where 39% of the intervention group participants had difficulty progressing through exercise training because of symptoms. A few even suggested less frequent sessions to allow for adequate recovery between activities. This highlights the need for individualized programs tailored to participants’ challenges to reduce the risk of symptom exacerbation.

After program completion, participants described challenges in sustaining exercise routines, often recommending a 4-week extension and peer-led group sessions to maintain engagement. These experiences underscore the challenge of transitioning from supervised rehabilitation to independent self-management. Literature supports strategies such as peer support, periodic check-ins, and digital tools to foster accountability, motivation, and confidence.^34^^,^^35^ Integrating ongoing support into primary care or community services may help sustain behavioral change and reduce the risk of relapse.^36^^,^^37^

Given the heterogeneous nature of long COVID and its association with PEM, the role of exercise often remains under discussion. This qualitative study advances the understanding of long COVID rehabilitation. Complementing the quantitative findings of the RCT,^8^ this study provides patient insights that inform an effective rehabilitation approach. While the RCT^8^ primarily focused on physical outcomes and symptoms, it was limited in addressing behavioral and relational aspects of recovery. This study highlights the importance of personalized support and growing self-efficacy, demonstrating the value of mixed-methods approaches in evaluating complex interventions for long COVID. Adding to these strengths, the study included both hospitalized and non-hospitalized participants and incorporated careful monitoring of adverse events, including those related to PEM, thereby enhancing its relevance and applicability.

### Study limitations

This study has limitations. First, control group participants were not interviewed, limiting contextual insight and causal interpretation. Second, while participants who initially consented to be interviewed were invited, response bias is possible, as those with strong opinions may have been more likely to participate. Finally, participant feedback on the findings was not collected, limiting validation.

## Conclusions

This study highlights the value of a flexible, individualized, virtual rehabilitation program for people with long COVID, particularly the importance of supervised, symptom-titrated exercise and education on pacing and symptom management. Future program design should prioritize ongoing support, longer durations, and structured post-rehabilitation maintenance strategies.

## Supplier

a. Dedoose, version 9.2; SocioCultural Research Consultants.

## Disclosure

J.B. reports financial relationships with AstraZeneca, the Canadian Institute of Health Research (CIHR), GSK, Novartis, Sanofi, Réseau en santé respiratoire (RSR) Fonds de recherche du Québec – Santé (FRQS), Fonds de recherche du Quebec (FRQ), Trudel, the McGill University Health Centre (MUHC) Foundation, American College of Chest Physicians (CHEST), Canadian Thoracic Society (CTS), Roche, Pfizer, Canadian Lung Association (CLA), and Respiplus. M.S.’s employer, Respiplus, a nonprofit society, has received sponsorship in the form of educational grants for the development of programs in the Coronavirus disease 2019 (COVID-19) field, including from AstraZeneca, GSK, Moderna, and Pfizer. R.Z. has a financial relationship with WillKin Health. The other authors have nothing to disclose.

## Appendix Supplementary materials

Image, application 1

Image, application 2

## Footnotes

Supplementary material associated with this article can be found, in the online version, at doi:10.1016/j.arrct.2025.100526.
