# Supplementary material for: Perspectives of Individuals With Long COVID on Virtual Physical Rehabilitation: A Qualitative Study
Source: Arch Rehabil Res Clin Transl. 2025 Sep 18;7(4):100526. doi: 10.1016/j.arrct.2025.100526 (PMC12750420; doi:10.1016/j.arrct.2025.100526)
Supplement: Supplementary file 1 [file mmc1.pdf]

Consolidated criteria for reporting qualitative studies (COREQ): 32-item checklist

No.	Item	Description	Section #
**Domain 1: Research team and reflexivity**			
Personal characteristics			
1.	Interviewer/facilitator	Which author/s conducted the interview or focus group?	Research Team and Reflexivity Page #4
2.	Credentials	What were the researcher's credentials? *E.g. PhD, MD*	
3.	Occupation	What was their occupation at the time of the study?	
4.	Gender	Was the researcher male or female?	
5.	Experience and training	What experience or training did the researcher have?	
Relationship with participants			
6.	Relationship established	Was a relationship established prior to study commencement?	Research Team and Reflexivity Page #4
7.	Participant knowledge of the interviewer	What did the participants know about the researcher? *E.g. Personal goals, reasons for doing the research*	
8.	Interviewer characteristics	What characteristics were reported about the interviewer/facilitator? *E.g. Bias, assumptions, reasons and interests in the research topic*	
**Domain 2: Study design**			
Theoretical framework			
9.	Methodological orientation and theory	What methodological orientation was stated to underpin the study? *E.g. grounded theory, discourse analysis, ethnography, phenomenology, content analysis*	Study design Page #4-5
Participant selection			
10.	Sampling	How were participants selected? *E.g. purposive, convenience, consecutive, snowball*	Participant selection Page #5
11.	Method of approach	How were participants approached? *E.g. faceto-face, telephone, mail, email*	
12.	Sample size	How many participants were in the study?	
13.	Non-participation	How many people refused to participate or dropped out? What were the reasons for this?	
Setting			
14.	Setting of data collection	Where was the data collected? *E.g. home, clinic, workplace*	Setting and Data

15.	Presence of nonparticipants	Was anyone else present besides the participants and researchers?	Collection Page #6
16.	Description of sample	What are the important characteristics of the sample? *E.g. demographic data, date*	Results: Patient characteristics Page #7 and Table 1
Data collection			
17.	Interview guide	Were questions, prompts, guides provided by the authors? Was it pilot tested?	Setting and Data collection Page #6
18.	Repeat interviews	Were repeat interviews carried out? If yes, how many?	
19.	Audio/visual recording	Did the research use audio or visual recording to collect the data?	
20.	Field notes	Were field notes made during and/or after the interview or focus group?	
21.	Duration	What was the duration of the interviews or focus group?	
22.	Data saturation	Was data saturation discussed?	
23.	Transcripts returned	Were transcripts returned to participants for comment and/or correction?	
**Domain 3: analysis and findings**			
Data analysis			
24.	Number of data coders	How many data coders coded the data?	Data analysis Page #6
25.	Description of the coding tree	Did authors provide a description of the coding tree?	
26.	Derivation of themes	Were themes identified in advance or derived from the data?	
27.	Software	What software, if applicable, was used to manage the data?	
28.	Participant checking	Did participants provide feedback on the findings?	
Reporting			
29.	Quotations presented	Were participant quotations presented to illustrate the themes / findings? Was each quotation identified? *E.g. Participant number*	Results Page #7 to #14 and Table 2
30.	Data and findings consistent	Was there consistency between the data presented and the findings?	Results and Discussion Page #7 to #17

31.	Clarity of major themes	Were major themes clearly presented in the findings?	Results Page #7 to #14
32.	Clarity of minor themes	Is there a description of diverse cases or discussion of minor themes?	
