# Supplementary material for: Perspectives of Individuals With Long COVID on Virtual Physical Rehabilitation: A Qualitative Study
Source: Arch Rehabil Res Clin Transl. 2025 Sep 18;7(4):100526. doi: 10.1016/j.arrct.2025.100526 (PMC12750420; doi:10.1016/j.arrct.2025.100526)
Supplement: Supplementary file 2 [file mmc2.pdf]

**INTERVIEW GUIDE**

**Introduction/purpose of the interview**
You have agreed to take part in an interview, which is a discussion with the research assistant about the virtual physical rehabilitation program that you have recently completed.

It will take about 30 minutes. Please answer all questions as fully and honestly as you can. The purpose of the interview is to help understand what you think about the virtual physical rehabilitation program that you participated in.

1. Do you have any questions?
2. Do you agree with this interview being recorded?

For the benefit of the tape, it is [date/time] and present there is [research assistant] and [patient's study number].

Before we start, I just have a few questions which you can answer yes or no to.

1. Do you understand what the interview is about?
2. Do you understand the purpose of the interview?
3. Are you happy to take part in the interview and be recorded?

**Withdrawal**
If anything, we speak about today does make you feel uncomfortable you are free to not discuss a particular topic, request for the recorder to be switched off to resume the interview after a short break or you can ask to terminate the interview all together at any point. If after this discussion has taken place, you wish to remove your comments from the study, please contact me, and none of your comments will be considered when performing the analysis.

**Questions related to the program**
Thank you for participating in the rehabilitation program offered by our team and for completing the questionnaire. We would now like to ask you some questions about the program. All information provided in this interview will be kept strictly confidential.

**Reason for participation, knowledge, and attitude**

- What was your reaction when you were offered participation in the virtual physical rehabilitation program?
- Did you have any concerns or questions? If yes, what were the questions or concerns? Did someone answer them?
- Why did you accept to participate in this rehabilitation program?
- Did you feel that the program would be beneficial for you?
- Did you feel that you had enough self-confidence, knowledge, and skills to perform the exercises at home?
- Did your family/friends support you participating in the rehabilitation program? Why?
- Will you continue to do the exercises recommended by the kinesiologist now that the program is finished?

- Did you feel that any of your baseline symptoms improved with the exercise program, other than shortness of breath, fatigue, and functional mobility?
- Do you intend to change your intentions/ habits concerning exercises following this program?

**Barriers**

- Did you encounter any barriers or challenges regarding the exercises?
- Do you feel the virtual session was a barrier to your exercise program?
- Did you have the necessary resources at home to do the program?
- Do you think it wouldn't have been possible for you to participate in the program if you didn't have a family member to help you during the sessions?
- Did you have any environmental stressors at home such as excessive noise, uncomfortable temperature, physical irritants, or lack of space?

**Feasibility**

- How was your experience with completing the exercises and questionnaires? (Probing questions: was it easy? Difficult? Stressing?)
- How was your experience with the live supervised sessions via Zoom? (Probing questions: was it easy? Difficult? Stressing?)
- Were you able to complete the independent sessions?
- How was your experience with the independent sessions? Did you like it or dislike it?
- How was your experience using the pulse oximeter during the exercises? (Probing questions: was it easy? Helpful? Difficult? Annoying?)
- How was experience with the monitoring of your saturation, intensity of exercises with Borg scale, heart rate during the exercise sessions?
- What did you think of the process for scheduling the sessions? (Probing questions: did it work well?)
- Did you have any difficulty in finding time to schedule the exercise sessions? Did the kinesiologists offer you flexible times?

**Delivery of the program**

- What did you like the most about the program?
- What did you dislike the most about the program? Why?
- What did you think of the type of exercises that were recommended to you?
- What did you think of the number of exercises that were recommended to you?
- What do you think about the ratio between supervised live sessions to independent sessions throughout the program?
- Did the exercise sessions make you too fatigued to carry out your usual daily activities?
- Did the exercise sessions give you energy to carry out your usual activities?
- Did the kinesiologist give you clear instructions and guidance on how to complete the exercises?
- Did you receive enough reinforcement/motivation during the program?
- Was the duration of the exercise sessions (40 minutes per session) good for you? Why?
- Was the frequency of three sessions per week ideal for you?
- What did you think of the total duration of the program of 8 weeks? Short/too long/ideal?

- Do you think this program would benefit other individuals living with long COVID? If yes/no, why?

**Safety**

- Did you feel safe performing the exercises with supervision of the therapist? Why?
- Did you feel safe performing the exercises without supervision? (Muscular/ Cardiovascular and Flexibility exercises)

**Educational session**

- What was your experience with the educational sessions?
- What did you learn from these sessions?
- What did you appreciate or dislike? Topics? Speakers? Format?
- Were there any more topics you would have liked to see included?
- How would you change or improve education for future participants?
- Would you recommend participating in this type of educational session to other individuals living with long COVID, friends, or colleagues?

**Modifications to the program/Recommendations**

- Are there any exercises or types of exercise that you think were not valuable for you? If yes, which one and why?
- Is there anything that is missing in the program?
- Are there any exercises or types of exercise that you think should have been added that were not? If yes, which one and why?
- Would you modify some parts of the activity? If yes, which one and why?
- Do you have some recommendations to improve the virtual physical rehabilitation program?

**Conclusion**

- Is there anything else you would like to say about the exercise program we did not cover in the previous questions?
